# Synergistic HDAC4/8 Inhibition Sensitizes Osteosarcoma to Doxorubicin via pAKT/RUNX2 Pathway Modulation

**DOI:** 10.3390/ijms26083574

**Published:** 2025-04-10

**Authors:** Anuja Gajanan Magar, Vivek Kumar Morya, Young-Ho Koh, Kyu-Cheol Noh

**Affiliations:** 1 School of Medicine, Hallym University, Chuncheon-si 24252, Republic of Korea; 2 Hallym University Dongtan Sacred Heart Hospital, Hwaseong-si 18450, Republic of Korea; 3 Ilsong Institute of Life Science, Hallym University, Seoul-si 14068, Republic of Korea; 4 Hallym University Sacred Heart Hospital, Anyang-si 14068, Republic of Korea

**Keywords:** osteosarcoma, combination therapy, apoptosis, anti-cancer, HDAC inhibitor, doxorubicin

## Abstract

Osteosarcoma is a highly aggressive bone malignancy, particularly challenging in metastatic cases, with a 5-year survival rate remaining under 30%. Although doxorubicin (doxo) is a standard first-line chemotherapeutic agent, its clinical utility is often hindered by the development of drug resistance and associated systemic toxicity. Emerging evidence highlights the role of epigenetic alterations, particularly those involving histone deacetylases (HDACs), in promoting chemoresistance. In this context, the present study aimed to evaluate the therapeutic potential of combining doxo with the selective HDAC inhibitors, tasquinimod (Tas, targeting HDAC4) and PCI-34051 (PCI, targeting HDAC8), in SJSA-1 osteosarcoma cells. Utilizing both 2D and 3D in vitro models, the combination treatment (referred to as the T4 group) significantly reduced cell viability by 57.69% in 2D cultures and decreased spheroid volume by 35.19% in 3D models. The apoptotic response was markedly enhanced, with late apoptosis reaching 64.59% and necrosis at 32.07%, both surpassing the effects observed with doxo alone. Furthermore, wound healing assays demonstrated a 37.74% inhibition of migration, accompanied by a decreased expression of the matrix metalloproteinases MMP9 and MMP13. Mechanistically, the combination therapy led to the downregulation of protein kinase B (pAKT) and RUNX2, along with upregulation of apoptotic markers, including caspase 8, caspase 3, and cleaved caspase 3, indicating a disruption of key survival pathways. These findings suggest that dual HDAC inhibition with Tas and PCI can potentiate doxo efficacy by enhancing apoptosis, inhibiting proliferation, and reducing metastatic potential, thus offering a promising strategy to overcome chemoresistance in osteosarcoma. Further preclinical and clinical studies are required to validate these therapeutic benefits.

## 1. Introduction

Osteosarcoma is a primary malignant bone tumor characterized by the production of immature bone, also known as osteoids, by mesenchymal cells. It is among the most common bone cancers, predominantly affecting adolescents and older adults (≥65 years) [1]. Osteosarcoma presents a significant clinical challenge owing to its high metastatic potential and recurrence rates. At diagnosis, approximately 20% of patients exhibit detectable metastases, whereas over 80% harbor micrometastases [2]. Historically, the 5-year survival rate for patients undergoing surgery alone was approximately 20%. However, the introduction of adjuvant chemotherapy and advancements in surgical techniques has improved survival rates to 60–70% [3]. Despite these advancements, survival outcomes have plateaued over the past three decades, with patients with metastatic osteosarcoma facing a dismal 5-year survival rate of less than 30%, emphasizing the urgent need for novel therapeutic strategies [3]. The current standard of care involves a multimodal approach, combining surgery with multi-agent chemotherapy, typically administered in neoadjuvant and adjuvant settings [4]. However, conventional chemotherapy often fails to provide durable responses due to chemoresistance, and high-dose regimens are associated with severe toxicities such as neutropenia, infections, and thrombocytopenia [3]. Thus, identifying alternative or adjunctive therapies that enhance efficacy while minimizing side effects is crucial [4].

One promising avenue involves combining doxorubicin (doxo), a first-line anthracycline chemotherapeutic agent, with histone deacetylase (HDAC) inhibitors [5,6]. Although doxo remains a cornerstone of osteosarcoma treatment, its clinical utility is hindered by dose-limiting toxicities, particularly cardiotoxicity, necessitating strategies to reduce cumulative doses without compromising efficacy [7]. Additionally, acquired resistance to doxo poses a major obstacle. Studies on doxo-resistant osteosarcoma cells have identified key molecular alterations that may serve as therapeutic targets to overcome resistance [7].

Epigenetic modifications, particularly those mediated by HDACs, play pivotal roles in the pathogenesis and chemoresistance of osteosarcoma. HDAC inhibitors restore normal gene expression by blocking HDAC enzymes, thereby sensitizing cancer cells to chemotherapy. In osteosarcoma, HDAC inhibitors can enhance doxo efficacy by promoting apoptosis, disrupting cell cycle regulation, and inhibiting DNA repair mechanisms. Notably, HDAC inhibitors downregulate DNA repair proteins, such as poly [ADP-ribose] polymerase 1, thereby impairing tumor cell survival under chemotherapeutic stress [5]. These findings suggest that targeting epigenetic pathways through HDAC inhibition could help overcome drug resistance and improve therapeutic outcomes [5,6]. Consequently, this study focused on the roles of HDAC4 and HDAC8 in osteosarcoma progression.

HDAC4 is a key regulator of gene transcription, cell proliferation, and cell survival. Its aberrant expression has been implicated in tumor progression across various cancers, and its overexpression is associated with higher tumor grades, advanced clinical stages, and poor prognosis. For instance, HDAC4 promotes metastasis in esophageal carcinoma and supports cell survival [8]. In osteosarcoma, HDAC4 is highly expressed and contributes to tumor development by modulating proliferation, invasion, and apoptosis [9]. However, paradoxically, its overexpression may counteract certain anti-cancer effects [10]. Despite these insights, the precise role of HDAC4 in osteosarcoma remains poorly defined, necessitating further investigation into its expression and function in osteosarcoma tissues. HDAC8 is increasingly recognized for its role in tumorigenesis. Elevated HDAC8 expression has been reported in hepatocellular carcinoma, where it promotes tumor growth and confers resistance to chemotherapy [11]. HDAC8 inhibition enhances sensitivity to doxo [12], whereas in colon cancer, HDAC8 represses pro-apoptotic genes and activates Jak2/Stat signaling [13]. It demonstrates anti-cancer effects by impairing melanoma cell proliferation and migration [14]. HDAC8 is involved in key oncogenic processes, including proliferation, metastasis, immune evasion, and drug resistance [15]. The combination of doxo with HDAC inhibitors offers a promising strategy to improve osteosarcoma treatment outcomes. By modulating epigenetic mechanisms, HDAC inhibitors can enhance chemotherapy sensitivity, inhibit tumor growth, and reduce metastatic potential. This approach not only aims to increase treatment efficacy but also seeks to mitigate the toxic side effects associated with high-dose chemotherapy. We hypothesize that combining doxo with tasquinimod (Tas), an HDAC4 inhibitor, and PCI, an HDAC8 inhibitor, will synergistically enhance apoptosis and suppress cell proliferation, even at reduced doxo doses. Specifically, we aim to restore doxo sensitivity in osteosarcoma cells by disrupting survival pathways and promoting apoptosis. To assess therapeutic potential, we will utilize the SJSA-1 osteosarcoma cell line, known for its aggressive phenotype, high tumorigenic potential, and metastatic capability, making it an ideal model for evaluating novel therapeutic strategies [16]. This study investigates the therapeutic potential of combining doxo with HDAC inhibitors, specifically Tas, an HDAC4 inhibitor, and PCI, a selective HDAC8 inhibitor, in osteosarcoma treatment. Given the significant role of HDACs in tumor progression, metastasis, and drug resistance, we hypothesize that targeting HDAC4 and HDAC8 will enhance doxo sensitivity, promote apoptosis, and inhibit tumor growth. By employing both 2D and 3D in vitro models using the aggressive SJSA-1 osteosarcoma cell line, we aim to elucidate the molecular mechanisms underlying this combination therapy, focusing on the protein kinase B (pAKT) and runt-related transcription factor 2 (RUNX2) signaling pathway. Through evaluating cell viability, apoptosis induction, migration inhibition, and molecular pathway alterations, this study seeks to provide compelling evidence that HDAC inhibition can overcome doxo resistance and enhance treatment efficacy. 

## 2. Results

### 2.1. Doxo and Combination Treatment Groups Inhibit the Cell Proliferation of the SJSA-1 Osteosarcoma Cell Line

**Figure 1:**
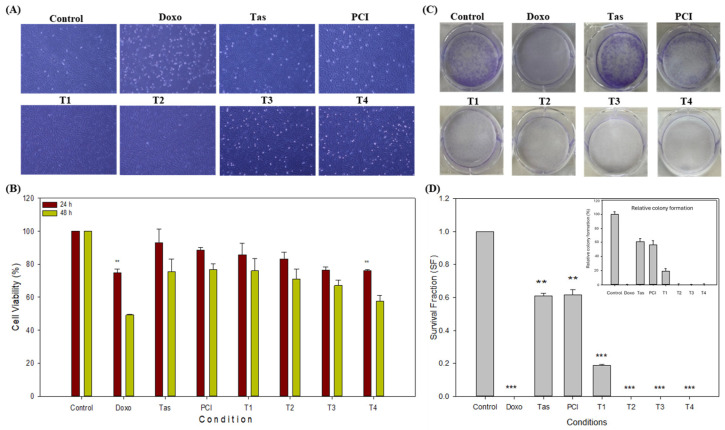
Inhibitory effects of doxorubicin (doxo), tasquinimod (Tas), and PCI-34051 (PCI), individually and in combination, on SJSA-1 osteosarcoma cell proliferation. (**A**) Representative microscopic images of SJSA-1 cells treated with doxo, Tas, and PCI alone and in combination for 48 h. Blue-colored areas indicate live cells, while white dots represent debris or dead cells. Changes in cell morphology demonstrate treatments’ effects on cell viability and proliferation (images captured at 4X magnification). (**B**) Cell viability (CCK-8 Assay): CCK-8 assay was performed after 48 h of treatment to evaluate viability of SJSA-1 cells. This assay measures metabolic activity as indicator of cell viability and cytotoxicity. Mean values and % errors are presented to compare cytotoxic effects of each treatment and combination therapy, showing their effectiveness in reducing cell viability. (**C**) Colony formation assay: long-term proliferative potential of SJSA-1 cells was assessed using colony formation assay. This assay quantifies cells’ ability to form colonies after treatment. Crystal violet staining (blue) was used to visualize colony confluency. A reduced intensity of blue staining indicates a lower number of colonies. Reduced colony numbers or smaller colony sizes indicate inhibitory impact on cell proliferation. (**D**) Quantification of colony formation assay: number of colonies was counted and analyzed to assess long-term proliferative capacity of cells. Bar graphs represent mean ± SD of colony count from three independent experiments. Statistical significance was determined using one-way ANOVA followed by Tukey’s post hoc test (** *p* < 0.01 and *** *p* < 0.001).

To systematically assess the inhibitory potential of doxorubicin (doxo), tasquinimod (Tas), and PCI-34051 (PCI) compounds, both as monotherapies and in combination, we utilized the SJSA-1 osteosarcoma cell line as a representative model. After 48 h of treatment, distinct morphological alterations, including cellular shrinkage and reduced cell density, were observed in the doxo, T3, and T4 treatment groups when compared to the control group. In contrast, no significant morphological changes were noted in the tas, PCI, T1, or T2 groups (Figure 1A).

The inhibitory concentration (IC50) of doxo in monotherapy was approximately 3 µM; however, in combination treatments, the IC50 decreased significantly, with the most pronounced reduction observed in the T4 combination group (0.5 µM doxo + 2 µM Tas + 2 µM PCI). The cell viability after 48 h was 49.26% in the doxo monotherapy group and 57.69% in the T4 combination group (Figure 1B). Notably, the doxo and T4 combination groups demonstrated comparable anti-proliferative effects within the 48 h timeframe. Additionally, the Tas (75.31%), PCI (76.61%), T1 (75.91%), T2 (71.01%), and T3 (67.10%) groups exhibited significant reductions in cell viability, indicating their intrinsic anti-proliferative effects. However, the most substantial reduction in cell proliferation was observed in the T4 combination group, suggesting a synergistic effect of histone deacetylase (HDAC) inhibitors that enhances the therapeutic efficacy of doxo while potentially mitigating cytotoxicity. A one-way ANOVA (Microsoft Excel 2016, Version 16.0.5495.1000, 64-bit) revealed a significant difference between the groups (*p* < 0.0001). Tukey’s HSD post hoc test confirmed that all treatment groups (doxo, Tas, PCI, T1, T2, T3, and T4) were significantly different from the control (*p* < 0.0001 for all comparisons) (Figure 1B). To ensure the robustness of our findings, we performed a CCK-8 assay using MG-63 osteosarcoma cell line. The T4 treatment group showed a cell viability of around 58.07% in the MG-63 cell line after 48 h (Supplementary Files, Figure S2). This cell line was derived from a different patient of a different ethnic background. The findings in the MG-63 cell line were consistent with those obtained in the SJSA-1 cell line, suggesting that our results are reproducible and not significantly affected by the ethnicity or genetic background of the cell line used. Therefore, we continued our further study using SJSA-1 cells.

To further evaluate the long-term effects of these treatments, a colony formation assay was conducted to assess the clonogenic potential of the SJSA-1 cells (Figure 1C). The colony formation assay results indicated a significant reduction in clonogenic survival following treatment. The plating efficiency (PE) of the control cells was calculated to be 23.42%, based on an initial seeding of 800 cells. Normalization of colony counts relative to the control allowed for the calculation of the survival fraction (SF) for each treatment condition. As expected, doxorubicin (doxo) treatment exhibited the most profound reduction, with a survival fraction close to zero, suggesting a near-complete inhibition of colony formation (Figure 1D). The Tas and PCI treatments maintained moderate clonogenic potential, with survival fractions of 0.610 and 0.615, respectively, indicating partial inhibition compared with the control. The experimental groups, T1, T2, T3, and T4 showed a progressive decline in clonogenic capacity, with T3 and T4 completely abolishing colony formation. This suggests a dose-dependent or mechanistic response that significantly affects osteosarcoma cell proliferation. These findings provide strong evidence for the efficacy of the tested treatments in reducing tumor cell survival and highlight the potential of combination approaches targeting osteosarcoma growth.

To assess the safety profiles of Tas and PCI, normal human tenocytes were treated with these compounds at the same concentrations used for osteosarcoma cells. Cell viability was evaluated using a CCK-8 assay. Importantly, no significant morphological changes, such as cell shrinkage or reduction in cell density, were observed following Tas or PCI treatment (Figure S1A). The cell viability in the doxo monotherapy group was 71.07%, whereas in the T4 combination group, it increased to 103.80% (Figure S1B). These findings confirmed that normal tenocyte viability remained largely unaffected in the combination treatment groups compared to doxo alone, suggesting that HDAC inhibitors selectively target osteosarcoma cells while sparing normal cells. This selective cytotoxicity underscores its potential as a safe adjunctive therapeutic agent for osteosarcoma treatment.

### 2.2. Combination Therapy with Doxo and HDAC Inhibitors Enhances Apoptotic Signaling in Osteosarcoma Cells

**Figure 2:**
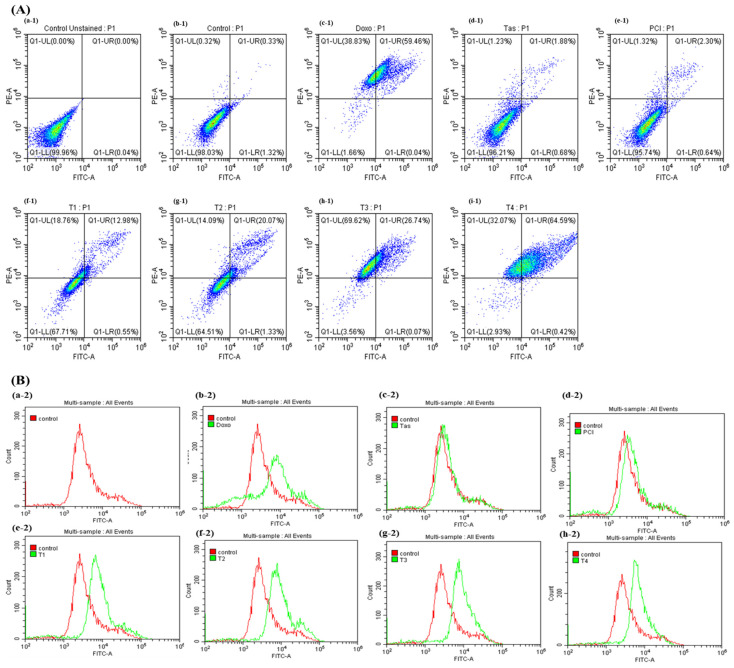
Effects of combination therapy of doxo, HDAC4 and HDAC8 inhibitors on apoptosis in osteosarcoma cells. (**A**) Rate of apoptosis in SJSA-1 osteosarcoma cells following 48 h of treatment with doxo, Tas, PCI, and combination therapies was assessed. This panel shows quantitative data on apoptosis induction, illustrating impact of each treatment alone and in combination on extent of apoptosis in osteosarcoma cells. (Treatment groups: (**a-1**)—unstained, (**b-1**)—control, (**c-1**)—Doxo, (**d-1**)—Tas, (**e-1**)—PCI, (**f-1**)—T1, (**g-1**)—T2, (**h-1**)—T3, (**i-1**)—T4.) (**B**) Caspase-3/7 activity was measured as indicator of apoptosis. Increased caspase activity points to higher apoptotic signaling, underscoring potential efficacy of combination therapy in promoting cell death in osteosarcoma cells. (Treatment groups: (**a-2**)—control, (**b-2**)—Doxo, (**c-2**)—Tas, (**d-2**)—PCI, (**e-2**)—T1, (**f-2**) –T2, (**g-2**)—T3, (**h-2**)—T4).

To elucidate the pro-apoptotic effects of combination therapy involving doxo and HDAC inhibitors, namely Tas and PCI, in osteosarcoma cells, flow cytometry was used to assess apoptosis induction. As illustrated in Figure 2A, treatment with the T4 regimen resulted in 64.59% of the cell population undergoing late apoptosis and 32.07% undergoing necrosis. In contrast, the doxo monotherapy group exhibited 38.83% necrosis and 59.46% late apoptosis. Notably, apoptosis was significantly elevated in the T4 treatment group compared to that in the doxo monotherapy group. Interestingly, HDAC inhibitors appeared to reduce necrosis while promoting late apoptosis, as observed in the T4 group, relative to the other combination therapy groups. This suggests that HDAC inhibition alone may exert a pro-apoptotic effect by modulating cell death.

To further investigate the molecular mechanisms underlying apoptosis induction, activation of caspase-3 and caspase-7, the key executioner proteases in both the intrinsic (mitochondrial) and extrinsic (death-receptor-mediated) apoptotic pathways, were analyzed by flow cytometry (Figure 2B). The results demonstrated a significant upregulation of caspase-3/7 activity in the combination therapy groups (T3 and T4) compared to both the control and doxo monotherapy groups. Notably, monotherapy with Tas (10 µM) and PCI (10 µM) also induced higher caspase activation than the doxo (IC50 = 3 µM) group, suggesting that HDAC inhibitors can independently initiate apoptotic cascades. However, the apoptotic effect was more pronounced when the HDAC inhibitors were co-administered with doxo, indicating a potential synergistic interaction.

Importantly, T4-treated cells exhibited the highest levels of apoptosis and caspase activation, surpassing the apoptotic induction observed in the monotherapy-treated cells. These findings indicate that the dual inhibition of HDAC4 and HDAC8 enhances apoptotic signaling, leading to increased cell death. The substantial upregulation of caspase-3/7 activity in response to combination therapy further supports the involvement of both the intrinsic (mitochondrial) and extrinsic (death receptor) apoptotic pathways. Collectively, these findings provide strong evidence that the synergistic interaction between doxo, Tas, and PCI promotes apoptosis in osteosarcoma cells through caspase activation. These results underscore the potential of HDAC-targeted combination therapy as a promising approach for overcoming chemoresistance and enhancing the therapeutic efficacy of osteosarcoma treatment.

### 2.3. Doxo and HDAC Inhibitors Combination Treatment Inhibits the Migration of Osteosarcoma Cells

**Figure 3:**
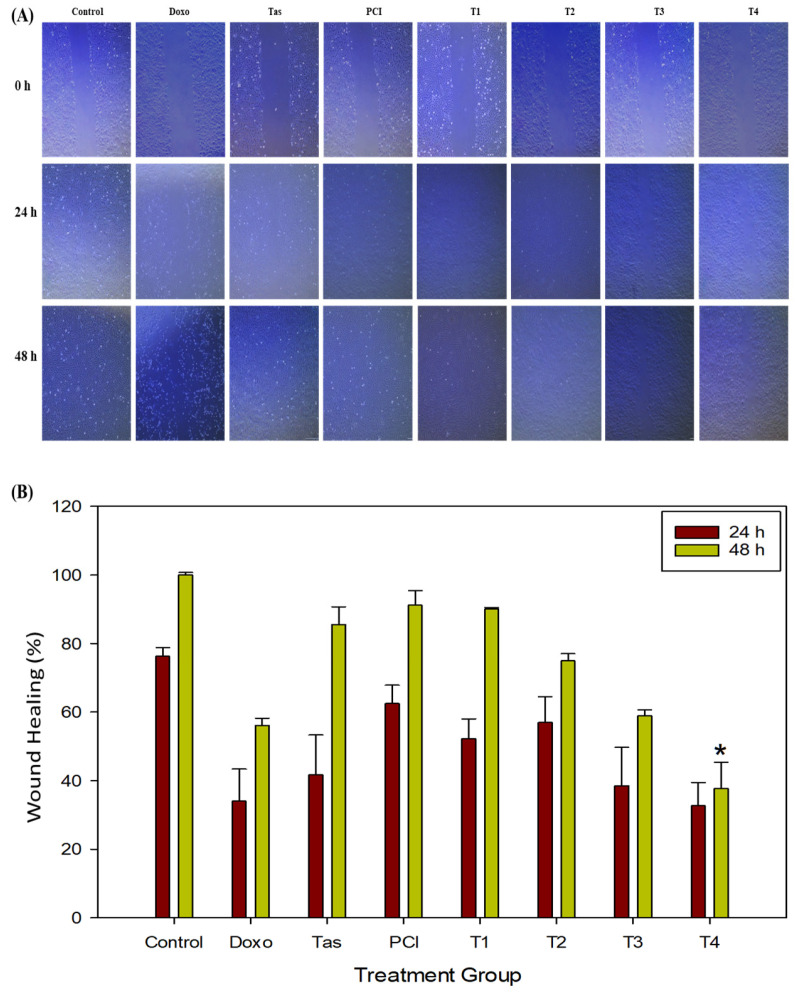
The effects of doxo, Tas, and PCI, individually and in combination, on the metastatic potential of SJSA-1 osteosarcoma cells. (**A**) Wound healing assay: images from a wound healing assay show the impact of doxo, Tas, PCI, and their combinations on the migratory ability of SJSA-1 cells. The degree of wound closure after 48 h of treatment illustrates the inhibitory effects of each treatment and combination therapy on cell migration, suggesting potential anti-metastatic properties (images captured at 4X magnification). (**B**) Quantification of wound healing (%) in SJSA-1 cells after 24 and 48 h of treatment. (* *p* < 0.05).

To evaluate the effects of doxo, Tas, and PCI on the migratory capacity of osteosarcoma cells, we conducted wound healing assays under various treatment conditions. Our findings showed that the T4 treatment group exhibited a modest reduction in wound closure compared to the doxo-treated group (Figure 3A,B). Specifically, after 24 h of treatment, the wound healing rate in the doxo-treated group was 34.01%, whereas the T4 treatment group exhibited a slightly lower rate of 32.73% (* *p* < 0.05). By 48 h, the wound closure rate in the doxo group increased substantially to 56.15%, whereas the T4 treatment group showed only a modest improvement, reaching 37.74%. These findings suggest that combination therapy (T4) effectively impaired the migratory ability of osteosarcoma cells. Additionally, while combination therapy (T4) appears to reduce osteosarcoma cell migration, the effect is modest and not clearly superior to that of doxo monotherapy.

In contrast, monotherapies with Tas (85.54%) and PCI (91.13%) had minimal effects on cell migration, indicating that HDAC inhibitors alone did not significantly alter cell motility. However, the co-administration of doxo appears to enhance its anti-migratory effects. A comparative analysis of the treatment groups further revealed that combination therapy (T4) resulted in a lower wound healing percentage compared to doxo monotherapy, suggesting that HDAC inhibitors may function as adjuvants by augmenting the anti-migratory and potentially anti-metastatic effects of doxo.

To further explore the molecular mechanisms underlying these observations, we examined the expression of key extracellular matrix (ECM)-degrading enzymes, matrix metalloproteinase (MMPs), particularly MMP9 and MMP13, which are crucial mediators of tumor invasion and metastasis. A Western blot analysis revealed marked downregulation of MMP9 and MMP13 protein levels in both the doxo and T4-treated groups compared to the control. In addition, MMP9 expression was downregulated in the T1, T2, and T3 combination groups. This suppression of MMP expression reinforces the hypothesis that combination therapy impairs osteosarcoma cell migration by inhibiting ECM degradation, potentially preventing metastatic dissemination.

### 2.4. Development of a 3D SJSA-1 Osteosarcoma Spheroid Model and Drug Screening of Doxo and HDAC Inhibitor Cocktail

**Figure 4:**
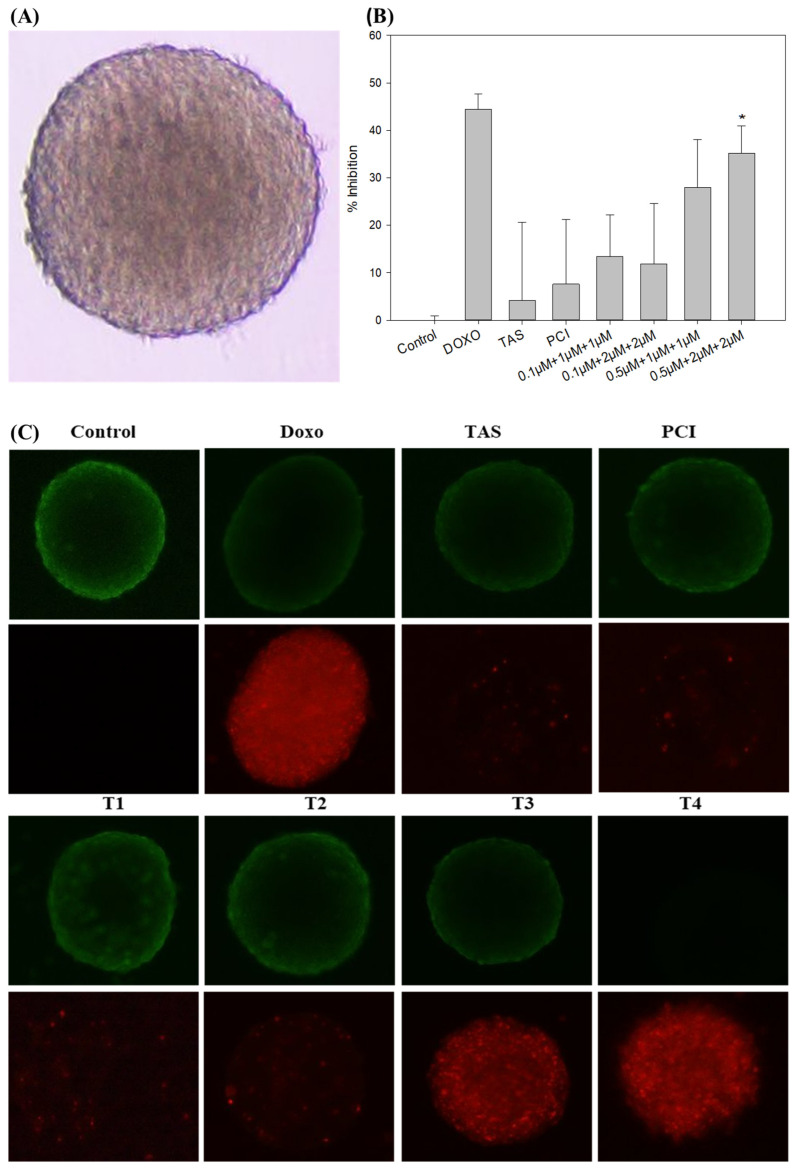
The evaluation of treatment effects on a 3D tumor spheroid model of SJSA-1 osteosarcoma cells. (**A**) Spheroid morphology: a representative image of the SJSA-1 3D osteosarcoma spheroid model taken using an Olympus IX73 microscope (magnification: 4X; scale bar: 500 µm) displays the spheroid structure before treatment, providing a baseline for assessing changes in response to treatment. (**B**) Spheroid volume: the average volume of SJSA-1 spheroids was measured after 48 h of treatment. Reduced spheroid volume indicates the treatment’s efficacy at limiting tumor growth within the 3D model. (**C**) Cytotoxicity assessment (LIVE/DEAD staining): the cytotoxic effects of doxo, Tas, PCI, and their combinations on the 3D spheroid model were assessed using calcein AM dye (live cells: green fluorescence) and propidium iodide dye (dead cells: red fluorescence) staining (magnification: 4X; scale bar: 500 µm). This staining highlights viable and non-viable cells within the spheroid, visually illustrating the differential cytotoxicity of each treatment approach. (* *p* < 0.05).

To better replicate the in vivo tumor microenvironment, we established a 3D osteosarcoma spheroid model using SJSA-1 cells. The model was developed using ultra-low attachment plates to facilitate cell aggregation and spheroid formation. Within 48 h, the SJSA-1 cells formed compact, well-defined spheroids with smooth surfaces, rounded morphologies, and high cellular densities (Figure 4A). This model was subsequently used to evaluate the therapeutic efficacy of doxo and HDAC inhibitors, both individually and in combination.

Following spheroid formation, drug treatments were administered for 48 h under conditions comparable to those in the 2D monolayer model. Quantitative analysis of spheroid size using ImageJ software version 1.54g (Java version: 1.8.0_345, NIH, Bethesda, MD, USA) demonstrated a dose-dependent reduction in spheroid dimensions following drug exposure. Monotherapy with Tas (4.19%) and PCI (7.58%) had minimal impact on spheroid size. In contrast, combination treatments exhibited significant cytotoxic effects, with doxo reducing spheroid size by 44.40% and the T4 combination by 35.19% (Figure 4B). The response observed in the doxo-treated group was comparable to that of the T4 combination group, suggesting an enhanced therapeutic effect through co-administration.

To further evaluate the impact of the treatment on spheroid viability, a fluorescence-based LIVE/DEAD viability assay was performed. This assay utilizes calcein AM, which fluoresces green in viable cells owing to intracellular esterase activity, and propidium iodide, which fluoresces red in cells with compromised plasma membrane integrity, indicative of apoptosis or necrosis. Untreated control spheroids exhibited strong green fluorescence, indicating high cell viability (Figure 4C). In contrast, spheroids treated with doxo, T3, and T4 displayed a significant increase in red fluorescence, suggesting widespread cell death. Notably, spheroids in the T4-treated group exhibited the highest level of apoptosis, reinforcing the hypothesis that the combination therapy exerts a synergistic cytotoxic effect.

These findings demonstrated the utility of the 3D SJSA-1 spheroid model as a physiologically relevant platform for drug screening. These results further suggest that while HDAC inhibitors alone have limited efficacy at reducing spheroid size, their combination with doxo significantly enhances tumor cell death. The observed increase in apoptosis in the combination treatment group suggests that HDAC inhibitors potentiate doxo efficacy, likely by modulating the key survival and apoptotic pathways. These findings highlight the potential of HDAC inhibitors as adjuvant agents in osteosarcoma treatment and warrant further mechanistic investigation to elucidate their role in overcoming osteosarcoma progression.

### 2.5. Molecular Mechanisms Underlying the Anti-Tumor Effects of Combination Therapy

To investigate the molecular mechanisms contributing to the anti-tumor effects of the combination therapy, we conducted a Western blot analysis to assess the expression levels of key oncogenic, metastatic, and apoptotic markers in SJSA-1 3D spheroids following various treatments. The observed reduction in osteosarcoma cell migration (Figure 3A,B) was corroborated by the molecular analysis of ECM-degrading enzymes, particularly MMP9 and MMP13. MMPs play a critical role in tumor cell invasion and metastasis by facilitating ECM remodeling and degradation. The Western blot analysis demonstrated a marked downregulation of MMP9 and MMP13 protein levels in the doxo and T4 treatment groups compared to the control. In addition, MMP9 expression was downregulated in the T1, T2, and T3 combination groups. Suppression of these MMPs further supports the hypothesis that combination therapy impedes osteosarcoma cell migration by inhibiting ECM degradation, thereby potentially limiting metastatic dissemination.

**Figure 5:**
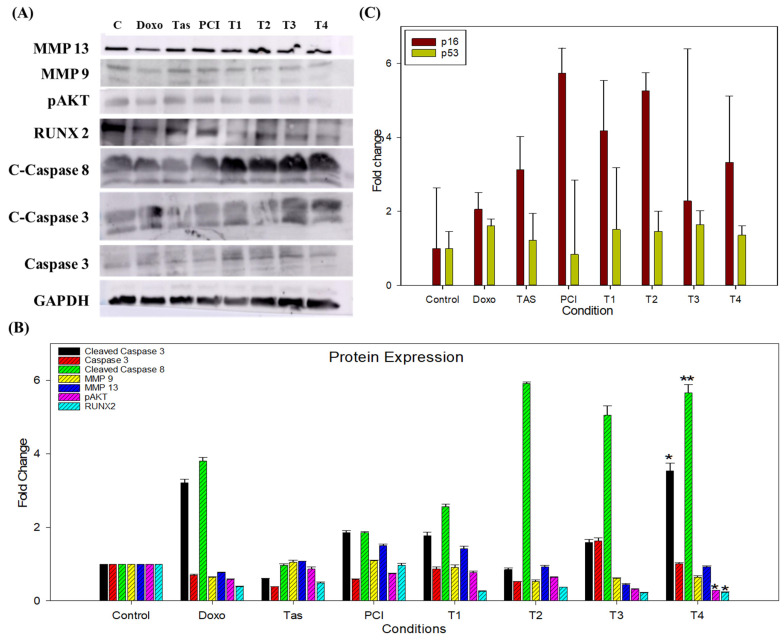
The impact of combination therapy on protein and gene expression in SJSA-1 osteosarcoma cells. (**A**) A Western blot analysis illustrating the effects of the combination treatment on key proteins involved in metastasis, cell survival, and apoptosis in SJSA-1 osteosarcoma cells. The expression levels of MMP9 and MMP13, which facilitate tumor cell invasion and metastasis through ECM degradation, were significantly reduced, indicating a potential inhibitory effect of the combination therapy on metastatic progression. Additionally, phosphorylated AKT (pAKT), a central regulator of the PI3K/AKT pathway associated with cell survival and chemoresistance, and runt-related transcription factor 2 (RUNX2), a critical modulator of osteosarcoma progression and tumor aggressiveness, were both downregulated, suggesting the suppression of key oncogenic pathways. Conversely, apoptotic markers, including cleaved caspase-8, cleaved caspase-3, and total caspase-3, exhibited increased activation following treatment, indicating enhanced apoptotic signaling. GAPDH was used as a loading control to ensure equal protein loading across samples. (**B**) A densitometric analysis of the Western blot bands representing the expression levels of target proteins after 48 h of treatment. The band intensities were quantified using Image Lab software (Version 6.1.0 build 7) and normalized to the GAPDH. Relative protein expression levels are expressed as a fold change compared to the untreated control group. The data represent the mean ± the standard deviation from two independent experiments. (**C**) qRT-PCR analyzed the gene expression of the apoptosis-related markers p16 and p53 following treatment. The results highlight the upregulation of p16 and stable expression of p53 in treated cells. (* *p* < 0.05 and ** *p* < 0.01).

The Western blot analysis revealed significant downregulation of phosphorylated protein kinase B (pAKT) and runt-related transcription factor 2 (RUNX2) in the T4 combination treatment group (Figure 5A,B). RUNX2, a well-established osteosarcoma biomarker, promotes tumor progression by enhancing cell proliferation, invasion, and resistance to apoptosis. Similarly, pAKT, a key regulator of the PI3K/AKT signaling pathway, plays a central role in cell survival, metabolism, and oncogenic transformation. The substantial suppression of these proteins in the T4-treated group suggests that the combination therapy effectively disrupted critical survival pathways, thereby impairing tumor growth and viability.

In addition to inhibiting oncogenic signaling, combination therapy induces robust apoptotic activation. The Western blot analysis demonstrated a pronounced increase in cleaved caspase-8, cleaved caspase-3, and total caspase-3 levels, with the most significant upregulation observed in T4-treated spheroids (Figure 5A,B). Notably, other combination treatments also exhibited increased caspase activation compared to the doxo monotherapy group. The activation of caspase-8, a hallmark of the extrinsic apoptosis pathway, suggests the involvement of death-receptor-mediated apoptosis, whereas elevated caspase-3 levels confirm the execution phase of apoptosis, leading to irreversible cellular disassembly. These findings highlight the potent pro-apoptotic effects of combination therapy, reinforcing its potential to effectively induce programmed cell death in osteosarcoma cells.

Gene expression analysis provided further insights into the molecular dynamics of the combination treatment. Notably, the PCI-treated group exhibited a significant 5.73-fold upregulation of cyclin-dependent kinase inhibitor 2A (p16), whereas the T2 and T4 groups displayed a 5.25-fold and 3.32-fold increase, respectively. In contrast, the doxo-treated group exhibited only a 2.05-fold upregulation relative to the control (Figure 5C). This substantial increase in p16 expression suggests that PCI may enhance the activation of this tumor suppressor, contributing to apoptosis induction and tumor growth inhibition.

Conversely, the expression of p53, a key tumor suppressor and regulator of apoptosis, remained largely unchanged, with only a 1.35-fold increase in the T4 combination group and a 1.60-fold increase in the doxo monotherapy group. This suggests that combination therapy may exert its anti-tumor effects through p53-independent mechanisms, potentially by modulating alternative apoptotic and cell cycle regulatory pathways.

Notably, the combination treatment groups exhibited a distinct molecular profile characterized by a significant reduction in osteosarcoma-associated markers, particularly RUNX2 and pAKT, while maintaining stable p53 expression levels. The concurrent suppression of these oncogenic proteins, coupled with the upregulation of apoptotic markers and p16, underscores the mechanistic synergy between HDAC inhibitors and doxo in targeting multiple tumor-promoting pathways. These findings provide compelling evidence that combination therapy effectively reprograms osteosarcoma cells toward apoptosis while disrupting key survival pathways, particularly the RUNX2/pAKT axis. This suggests a promising therapeutic strategy for overcoming chemoresistance and enhancing treatment efficacy in osteosarcoma.

## 3. Discussion

Doxorubicin (doxo) remains a cornerstone chemotherapeutic agent for osteosarcoma because of its potent cytotoxic effects, which are primarily mediated by DNA intercalation and the inhibition of nucleic acid synthesis. Additionally, doxo disrupts mitochondrial function by targeting the electron transport chain and oxidative phosphorylation, leading to increased oxidative stress and apoptosis in cancer cells [7,17]. However, despite its efficacy, the clinical application of doxo is significantly hindered by dose-dependent cardiotoxicity and the emergence of drug resistance, particularly in pediatric patients. This necessitates the development of novel therapeutic strategies that enhance anti-tumor efficacy while minimizing adverse effects [7]. In this context, the combination of doxo and histone deacetylase (HDAC) inhibitors represents a promising avenue for optimizing osteosarcoma treatment by targeting complementary oncogenic pathways. To contextualize our findings, we compiled a summary of doxorubicin-based combination strategies reported in the literature (Table 1), highlighting the approaches to enhance doxo efficacy through synergistic mechanisms.

HDACs are critical regulators of key cellular processes, including cell cycle progression, apoptosis, and metastasis, making them attractive therapeutic targets in oncology. Among the HDAC family, HDAC4 and HDAC8 have gained significant attention because of their distinct roles in tumor biology [10,18]. HDAC4 has been implicated in osteosarcoma progression by promoting chemoresistance and enhancing cell survival, underscoring its relevance as a therapeutic target [10]. In contrast, HDAC8 has been shown to facilitate tumor proliferation, metastasis, and immune evasion in multiple cancer types, although its precise role in osteosarcoma remains unclear, necessitating further investigation [11,12,13].

Given these considerations, this study aimed to evaluate the therapeutic potential of targeting HDAC4 and HDAC8 using their specific inhibitors, tasquinimod (Tas) and PCI-34051 (PCI). Both Tas and PCI have demonstrated potent anti-cancer activity in preclinical models, with Tas effectively reducing cancer cell invasiveness and metastatic progression, leading to improved survival outcomes [20]. Meanwhile, PCI, a highly selective HDAC8 inhibitor, exhibits remarkable specificity, with a 4200-fold preference for HDAC8 over other HDACs, making it a compelling candidate for targeted therapy [11,12,13]. While broad-spectrum HDAC inhibitors, such as romidepsin and panobinostat, have been shown to exert anti-proliferative effects against osteosarcoma [21], the specific application of Tas and PCI, particularly in combination with doxo, remains largely unexplored. Thus, this study systematically assessed the anti-tumor efficacy of doxo, Tas, and PCI, both individually and in combination, to determine their potential for synergistic interactions in enhancing osteosarcoma treatment, and it has demonstrated that the combination of doxo with Tas and PCI exerts a synergistic inhibitory effect on osteosarcoma cell proliferation, as evidenced by a pronounced reduction in cell density, morphological alterations, and increased cell death. Microscopic examination of the treated cells revealed significant changes, including cellular shrinkage and detachment, particularly in the combination treatment groups. Functional assays further substantiated these observations. While neither Tas nor PCI alone significantly inhibited osteosarcoma cell proliferation, their combination with doxo markedly enhanced anti-tumor activity, as demonstrated by the CCK-8 assay. The colony formation assay provided additional support, showing a substantial reduction in colony-forming potential, with the T3 and T4 treatment groups exhibiting complete inhibition of colony formation, comparable to that of doxo monotherapy. The pronounced decline in colony numbers, particularly in the doxorubicin (doxo), T2, T3, and T4 groups, suggests a strong cytotoxic effect, leading to the near-complete inhibition of long-term cell survival. This is consistent with previous results indicating that chemotherapeutic agents and epigenetic modulators, such as HDAC inhibitors, can suppress osteosarcoma proliferation by disrupting key survival pathways. Interestingly, the Tas and PCI treatments retained a moderate colony-forming ability, indicating a partial inhibitory effect, which could be linked to mechanisms such as residual RUNX2/PI3K/AKT signaling or metabolic adaptation. The significant reduction in clonogenic survival at T1 and T2 further supports the hypothesis that targeted interventions affecting both proliferation and survival signaling pathways could enhance therapeutic efficacy. These findings highlight the potential of combination strategies, particularly those involving HDAC inhibitors and metabolic modulators, to improve osteosarcoma treatment outcomes.

Interestingly, PCI demonstrated superior anti-proliferative efficacy compared to Tas, consistent with previous studies indicating PCI’s ability to enhance acetyl-p53 (K381) in a dose-dependent manner, thereby suppressing tumor growth [18]. Our results align with the existing literature suggesting that selective HDAC inhibitors sensitize osteosarcoma cells to chemotherapy by reducing proliferation and inducing apoptosis [5]. Importantly, our cytotoxicity assessment in normal human cells indicated that Tas and PCI exerted minimal toxicity, with no significant effects on cell viability or morphology. Notably, Tas and PCI appeared to promote tenocyte growth, suggesting their potential biocompatibility and safety for therapeutic applications (Supplementary Figure S1A,B).

Apoptosis, a fundamental mechanism for maintaining cellular homeostasis, is tightly regulated through intrinsic and extrinsic pathways, both of which converge on the activation of caspase-3, a key effector of programmed cell death [22]. The extrinsic apoptotic pathway, mediated through caspase-8 activation, plays a crucial role in Fas-associated death domain recruitment and formation of the death-inducing signaling complex [23].

Previous studies have demonstrated that HDAC inhibition enhances chemotherapy-induced apoptosis by modulating apoptotic signaling cascades. Specifically, silencing HDAC4 has been shown to potentiate cisplatin-induced apoptosis by upregulating pro-apoptotic genes such as BIK in a p53-dependent manner [24]. Additionally, PCI, when combined with other agents such as ACY-241, suppresses cell proliferation, promotes apoptosis, and downregulates key anti-apoptotic and metastasis-associated proteins [18]. Similarly, targeted HDAC8 inhibition sensitizes cancer cells to chemotherapy by suppressing the multidrug resistance gene MDR1 [11].

Consistent with these findings, our study demonstrated that the combination of Tas and PCI significantly increased the levels of cleaved-caspase-3 and cleaved-caspase-8 in the T3 and T4 treatment groups, indicating the activation of both intrinsic and extrinsic apoptotic pathways. Caspase-3/7 activity was markedly elevated in these treatment groups, reinforcing the role of HDAC inhibition in facilitating apoptosis via mitochondrial and death-receptor-mediated mechanisms. Notably, this combination therapy exhibited minimal cytotoxicity to normal human cells, further supporting its therapeutic potential.

Metastasis remains a major challenge in osteosarcoma treatment, with epithelial–mesenchymal transition (EMT) playing a pivotal role in tumor invasion and dissemination. MMPs, particularly MMP9 and MMP13, facilitate ECM degradation, thereby promoting metastatic progression. HDAC4 has been identified as a key regulator of EMT, and its overexpression enhances tumor invasiveness in various cancers [25]. Similarly, HDAC8 has been implicated in colorectal cancer metastasis and melanoma brain invasion [26].

In our study, Tas and PCI significantly suppressed MMP9 and MMP13 expression, leading to reduced osteosarcoma cell migration and invasion, as evidenced by the wound healing assays. These effects were particularly pronounced in the T3 and T4 treatment groups, reinforcing the potential of combination therapy to mitigate metastasis.

To better model the tumor microenvironment, we employed a 3D spheroid system that closely recapitulates the in vivo tumor architecture and cellular interactions [27]. Our results demonstrated that combination therapy effectively reduced spheroid volume, suggesting enhanced tumor penetration and cytotoxicity. LIVE/DEAD cell staining further confirmed the increased cell death in the combination treatment groups, consistent with our 2D findings. However, due to the scope and timeline of the current study, we did not perform a dedicated spheroid regrowth/recovery experiment after the 48 h treatment period. Our primary focus was on evaluating the initial cytotoxic and migrastatic effects of the drug treatments within a defined timeframe. We followed the standard protocol for drug screening described in previously published research [28,29].

The phosphatidylinositol 3-kinase/protein kinase B (PI3K/AKT) signaling pathway, a crucial regulator of tumor proliferation and drug resistance, was significantly downregulated following combination therapy [30]. HDAC8 stabilizes Akt by reducing its acetylation, thereby enhancing its phosphorylation at Ser473, a process that promotes cell survival and chemoresistance [30]. Our study demonstrated that Tas and PCI reduced the expression of phosphorylated AKT (p-AKT) and runt-related transcription factor 2 (RUNX2), thereby disrupting these oncogenic pathways. RUNX2 overexpression is a hallmark of osteosarcoma and contributes to p53 suppression, leading to reduced responsiveness to chemotherapy. Meanwhile, our findings suggest that downregulation of RUNX2/pAKT signaling may restore p53 activity and sensitize osteosarcoma cells to apoptosis [19,30,31]. Further studies, such as siRNA knockdown or overexpression experiments, are needed to confirm a direct causal relationship. Additionally, emerging evidence underscores the significance of HDAC inhibitors in modulating osteosarcoma progression. Recent studies have demonstrated that HDAC inhibitors, such as romidepsin, can effectively downregulate neuropilin-1 (NRP1) in osteosarcoma cells, thereby significantly reducing their metastatic potential [32]. In the context of oncogenic signaling, the reciprocal activation between RUNX2 and the PI3K/AKT pathway has been recognized as a pivotal factor in driving tumor progression and fostering chemoresistance in osteosarcoma. the reciprocal activation between RUNX2 and the PI3K/AKT pathway has been recognized as a pivotal factor in driving tumor progression and fostering chemoresistance in osteosarcoma. RUNX2 plays a central role in the epithelial–mesenchymal transition (EMT), a critical process facilitating cancer cell migration and invasion, which ultimately promotes tumor progression. Multiple signaling pathways, including the PI3K/AKT pathway, have been reported to mediate the role of RUNX2 in cancer invasion [33]. Moreover, studies have shown that miR-302b can suppress osteosarcoma cell migration and invasion by directly downregulating RUNX2 expression, further reinforcing the importance of this pathway in osteosarcoma pathogenesis [34]. By integrating these insights, our study highlights the therapeutic potential of HDAC inhibitors in osteosarcoma treatment. Modulation of NNMT-SIRT1 interactions, optimization of combination therapies, and targeted inhibition of RUNX2-driven signaling pathways could serve as valuable strategies to enhance treatment efficacy and overcome chemoresistance in osteosarcoma.

However, recent studies have highlighted the critical role of NNMT in osteosarcoma progression, demonstrating that its upregulation contributes to increased tumor aggressiveness and resistance to therapy [35]. NNMT catalyzes the methylation of nicotinamide, reducing its availability as an inhibitor of SIRT1, a key regulator of cellular metabolism and stress responses [36]. Given that SIRT1 has been implicated in the modulation of apoptosis, DNA repair, and chemotherapy resistance, the interplay between NNMT and SIRT1 may significantly influence osteosarcoma cell survival. Our findings suggest that targeting HDACs in osteosarcoma, particularly in combination with agents that modulate metabolic pathways, could be further optimized by considering NNMT-SIRT1 interactions. Since NNMT-driven SIRT1 activation could potentially counteract HDAC inhibition, future studies should explore whether NNMT inhibitors could enhance the therapeutic efficacy of HDAC inhibitors in osteosarcoma treatment.

Although these findings underscore the potential of HDAC inhibitors in osteosarcoma therapy, several limitations remain. The long-term efficacy of this therapeutic strategy requires further validation in in vivo models, particularly concerning pharmacokinetics, toxicity, and potential resistance mechanisms. Combination therapies, such as doxo with PCI-24781, have shown promising anti-tumor responses with minimal toxicity in preclinical models [5], reinforcing the need for further investigation.

The results indicate that combination therapy (T4) effectively disrupted osteosarcoma cell migration. However, we acknowledge the absence of an isobolographic analysis, which is crucial for quantitatively determining whether the observed effects are synergistic, additive, or sub-additive. Future studies should incorporate dose–response matrix analyses and combination index calculations to precisely characterize the nature of drug interactions. Additionally, exploring a range of dose ratios will provide a clearer understanding of optimal treatment strategies.

To facilitate clinical translation, future research should focus on optimizing dosing regimens, determining whether a combination should be administered simultaneously or sequentially, and identifying biomarkers for patient stratification. These steps are essential for tailoring the treatment to individual patient responses.

## 4. Materials and Methods

### 4.1. Cell Culture

The human osteosarcoma cell lines SJSA-1 (CRL-2098) and MG-63 (CRL-1427) were obtained from the American Type Culture Collection (ATCC). The cells were cultured in Dulbecco’s Modified Eagle’ Medium (DMEM F12; SolBio; Gyeonggido, Republic of Korea) supplemented with 10.0% fetal bovine serum (FBS; Gibco; Thermo Fisher Scientific, Waltham, MA, USA) and 1.0% penicillin–streptomycin (Gibco; Thermo Fisher Scientific, Waltham, MA, USA). The cells were maintained in a humidified incubator at 37 °C and 5.0% CO_2_. Cells at passages 4 to 6 were used for the experimental procedures.

### 4.2. Drug Treatment Groups

This study investigated the effects of HDAC4 inhibitor (Tas) (MedChemExpress; Monmouth Junction, NJ 08852, USA; cat. HY-10528), and HDAC8 inhibitor (PCI) (MedChemExpress; Monmouth Junction, South Brunswick, NJ, USA; cat. HY-15224), and Doxo (Abcam; Cambridge, UK; cat. ab232855) in the SJSA-1 osteosarcoma cell line and spheroid model, both individually and in combination. In brief, eight treatment groups were included in this study (Table 2). Treatments were administered for 48 h in both 2D and 3D spheroid models with incubation at 37 °C and 5.0% CO_2_. The inhibitors Tas and PCI were received in powder form and dissolved in DMSO according to the manufacturer’s instructions to prepare 10 mM stock solutions, which were stored at −80 °C. Doxorubicin was received as a 20 mM stock solution and was directly diluted in the appropriate growth medium for treatments. For all experiments, the stock solutions were further diluted in complete growth medium to achieve the required final concentrations. Drug concentrations and treatment exposures were selected based on previous studies. As the aim was to evaluate the drugs in combination, the concentration of each drug was reduced compared to the doses typically used in monotherapy. This strategy minimizes potential toxicity while maximizing synergistic effects, allowing each drug to exert its intended mechanism of action while reducing adverse interactions. The 48 h treatment duration was optimized based on cell viability assay data, as it showed almost 50.0% inhibitory effects on osteosarcoma cell lines within this time span. All molecular assays were performed after 48 h treatment.

### 4.3. CCK-8 Assay

The CCK-8 assay is a colorimetric method that is used to gauge the metabolic activity of viable cells. It involves the reduction of WST-8 by mitochondrial dehydrogenases, culminating in the formation of a highly water-soluble yellow formazan compound [6]. The CCK-8 assay was performed using a CCK-8 cell counting kit (Nanjing Vazyme Biotech; Jiangsu, China; cat. A311) according to the manufacture’s protocol. Briefly, a cell suspension containing 5000 SJSA-1 osteosarcoma cells per well was seeded into a 96-well plate in a final volume of 100.0 μL. The cells were incubated overnight. The growth medium was then replaced with the drug treatment medium, and the cells were incubated further for 24 and 48 h at 37 °C. After incubation, the cells were washed with phosphate-buffered saline (PBS). Next, 100.0 µL of fresh complete growth medium and 10.0 µL of CCK-8 reagent were added to each well, and the plate was incubated for an additional 2 h at 37 °C, following the manufacturer’s protocol (Vazyme A311). The absorbance was measured at 450 nm using a microplate reader (BioTek Instruments, Inc., Winooski, VT, USA; S/N.1503205). The experiment was performed in triplicate (*n* = 3) with three independent biological replicates to ensure reproducibility. The total colony number was measured, and the results were normalized to the control group. Plating efficiency (PE) and surviving fraction (SF) were calculated based on the initial seeding density. Statistical analysis was performed to assess differences between the treatment groups.

### 4.4. Colony Formation Assay

SJSA-1 cells were plated in 6-well plates at a density of 8 × 10^2^ cells per well. After 24 h of incubation, the cells were treated with varying drug concentrations and cultured for an extended period. Once visible colonies appeared in the control group, the cultures in all treatment groups were harvested. The cells were washed with PBS, fixed in 4.0% paraformaldehyde (Sigma-Aldrich; Merck KGaA, Darmstadt, Germany) for 10 min, and then stained with crystal violet (Abcam) to facilitate colony visualization.

### 4.5. Annexin-V FITC Apoptosis Assay

Apoptosis in osteosarcoma cells was analyzed by flow cytometry using the Annexin-V FITC and PI staining kit (Abcam ab14085). SJSA-1 osteosarcoma cells were seeded in 60 mm culture plates and incubated until they reached 70.0–80.0% confluence. The medium was then replaced with the drug treatment medium. After 48 h of incubation, cells were washed with cold PBS, harvested using 0.25X trypsin (SolBio; Gyeonggido, Republic of Korea), and cell viability was assessed. The cells were subsequently suspended in 500.0 µL of binding buffer (R&D Systems; Minneapolis, MN, USA) and incubated in the dark with 5.0 µL of annexin V-FITC and 5.0 µL of propidium iodide, following the manufacturer’s instructions (Abcam). Flow cytometric analysis was performed using a Beckman Coulter CytoFlex flow cytometer (Beckman Coulter Inc., Suzhou, China) with detection filters set at 530/30 (annexin-V FITC) and 610/20 (PI), using an excitation laser at 488 nm.

### 4.6. Caspase-3/7 Activity Assay

Caspase-3/7 activity was measured using the CellEvent™ caspase-3/-7 Green Detection Reagent (Invitrogen; Life Technologies corporation, Eugene, OR, USA). Upon cleavage by activated caspase-3/7, the probe became fluorescent and free to bind to DNA. After 48 h of treatment, cells were collected and incubated with 5 μM CellEvent™ caspase-3/7 green detection reagent in complete medium for 30 min at 37 °C in the dark. The stained cells were observed using a Beckman Coulter CytoFlex flow cytometer (Suzhou, China).

### 4.7. Wound Healing Assay

SJSA-1 cells (0.5 × 10^6^ cells per well) were seeded into 6-well plates. Once the cells reached near confluence, a linear artificial wound was generated using a sterile 200.0 μL pipette, perpendicular to the cell layer. After gently washing with PBS, the cells were incubated with drug-containing medium for 48 h. Following incubation, the cells were fixed by adding 500.0 μL of fixative solution to each well and left to fix for 10 min at room temperature. The solution was aspirated, and the wells were carefully washed with PBS. Finally, 1.0 mL PBS was added to each well to maintain cell hydration. At 0, 24, and 48 h, each wound was randomly photographed (Olympus, IX73; Tokyo, Japan) and the percentage wound healing rate was calculated.

### 4.8. Spheroid Development

A 3D osteosarcoma spheroid model was established using a 96-well ultra-low attachment plate (Nunclon™ Sphera™ 96-Well; Thermo Fisher Scientific; Waltham, MA, USA). When SJSA-1 osteosarcoma cells reached 70.0–80.0% confluence, the cell culture medium was aspirated, and the cells were washed with 1X PBS. The cells were then trypsinized, and cell density was determined using trypan blue and a hemocytometer. Subsequently, 5000 cells were seeded in each well of a 96-well ultra-low-attachment plate containing 200.0 µL of complete growth medium. The plates were centrifuged at 1250 rpm for 5 min at 4 °C to promote cell aggregation at the center. After centrifugation, the plates were incubated for 48 h at 37 °C, 5.0% CO_2_, and 95.0% humidity [28,37]. Spheroids were generated after 24 h of incubation, whereas a more spherical and compact spheroid shape emerged after 48 h of incubation, which was used for drug screening.

### 4.9. Spheroid Size Analysis

The size of SJSA-1 spheroids was measured using the open-access software ImageJ version 1.54g (Java version: 1.8.0_345, NIH, Bethesda, MD, USA). In brief, the spheroid was converted into a binary mask using an image processing tool. The major and minor axes of the spheroid binary mask were determined using an analysis tool, and spheroid volume was calculated. Furthermore, the effect of treatment was evaluated through %inhibition using the following formula:

$$\%\mathrm{Inhibition}=\frac{Avg.\;Volume\;of\;control\;spheroid-Volume\;of\;treated\;spheroid\times 100}{Avg.\;Volume\;of\;control\;spheroid}$$

### 4.10. Live and Dead Cell Assay

After 48 h of treatment, the viability of the spheroid model was assessed using the LIVE/DEAD Cell Viability Assay Kit for 3D and 2D cell cultures (Sigma-Aldrich; Merck KGaA, Darmstadt, Germany). In brief, staining solution was prepared by mixing calcein AM and propidium iodide in a 1:1 ratio in mixture of complete growth medium and PBS (50:50 ratio). The cell culture medium was removed, and the spheroids were washed with 200.0 µL of PBS. Then, 100.0 µL of the staining solution was added to each well. Spheroids were incubated for 60 min at 37 °C, protected from light. Live and dead cells were visualized using a fluorescence microscope with the appropriate fluorescence filters for calcein AM (green fluorescence) and propidium iodide (red fluorescence) using Olympus IX73 microscope (Olympus Corporation, Tokyo, Japan).

### 4.11. Western Blot

After 48 h of treatment, the spheroids were collected from a 96-well plate into 1.5 mL microcentrifuge tubes. The spheroids were washed with PBS, followed by the addition of 100.0 µL of RIPA lysis buffer (ThermoScientific, Rockford, IL, USA; Ref. 89900), and protein concentration was determined using a BCA kit (Vazyme, cat. E112-02). The samples were then placed in an icebox for 120 min and vortexed every 10–15 min. After incubation, the samples were centrifuged at 14,000 rpm for 10 min, and the supernatant was collected and stored at −20 °C. After protein isolation, samples were mixed with a 5× sample buffer and subjected to SDS-PAGE on a polyacrylamide gel. The proteins were transferred to a PVDF membrane (Atto, Amherst, NY, USA, AE-667-P) and blocked with 5.0% BSA at room temperature for 1 h. The membranes were then incubated overnight at 4 °C with primary antibodies as follows: cleaved caspase 8 (Cell Signaling, Danvers, MA, USA, cat.98134S, 1:1000), caspase 3 (Cell Signaling, cat.9662S, 1:1000), cleaved caspase 3 (Cell Signaling, cat.9664S, 1:1000), RUNX2 (Cell Signaling, cat.12556S, 1:1000), MMP9 (SantaCruz, Dallas, TX, USA, cat.sc-21733, 1:1000), MMP13 (SantaCruz, cat.515284, 1:1000), pAKT (Cell Signaling, cat.4060S, 1:1000), GAPDH (R&D Systems, cat. MAB5718, 1:1000). Following incubation, the membranes were washed three times with 0.1% TBST for 5 min each and then incubated with HRP-conjugated goat anti-rabbit IgG (SantaCruz, cat.sc-2357, 1:1000) or anti-mouse IgG (R&D Systems, cat.HAF007, 1:1000) secondary antibodies for 2 h at room temperature. This washing step was repeated to remove excess secondary antibodies. Protein bands were visualized using an ECL detection kit (Vazyme) and recorded using a ChemiDoc Image Analyzer (GE Healthcare Life Sciences AB; Uppsala, Sweden; Amersham Imager 600 UV,). Densitometric analysis of the bands was performed using the software ImageLab (Version 6.1.0 build 7). Band intensities were normalized to GAPDH, and the fold change was calculated relative to the control group.

### 4.12. Real-Time Quantitative PCR (RT-qPCR)

RNA was extracted from spheroids using TRIazol Reagent (Ambion Life Technologies, Waltham, MA, USA, Ref. 15596026). Briefly, 1.0 mL of TRIazol reagent and 200.0 µL of chloroform were added to the samples, and the tubes were vortexed vigorously for 5–10 s. The tubes were then incubated at room temperature for 2–3 min before centrifugation at 12,000× *g* for 15 min at 4 °C. The colorless upper phase containing RNA was transferred to a fresh, RNase-free tube, and an equal volume of 100.0% ethanol was added to achieve a final ethanol concentration of 50.0%. The samples were mixed well by vortexing. RNA concentration and purity were assessed using a microplate reader (BioTek Instruments, Inc.; Winooski, VT 05404, USA, S/N.1503205). RNA was normalized and reverse-transcribed using the Vazyme cDNA synthesis kit (R312-02 HiScript III 1st Strand cDNA Synthesis Kit), according to the manufacturer’s instructions. cDNA synthesis was performed using a SimpliAmp Thermal Cycler (Applied Biosystems; Life Technologies; Carlsbad, CA, USA; S/N. 1708). The cDNA was then subjected to quantitative real-time PCR using SYBR Green Premix (Vazyme Taq Pro Universal SYBR qPCR Master Mix Q712; Nanjing Vazyme Biotech; Jiangsu, China) and analyzed on a LightCycler 96 (Roche, Basel, Switzerland). The RT-qPCR conditions were as follows: initial denaturation at 95 °C for 30 s, followed by 40 cycles of amplification at 95 °C for 10 s and 60 °C for 30 s, with a melting curve step at 95 °C for 15 s, 60 °C for 60 s, and 95 °C for 1 s, ending with a 30 s cooling step at 37 °C. The primer pair’s p16 (forward sequence: 5′-CTCGTGCTGATGCTACTGAGGA-3′; reverse sequence: 5′-GGTCGGCGCAGTTGGGCTCC-3′) and p53 (forward sequence: 5′-CCTCAGCATCTTATCCGAGTGG-3′; reverse sequence: 5′-TGGATGGTGGTACAGTCAGAGC-3′) were sourced from Origene Biotechnology, and β-actin (forward sequence: 5′-CACCATTGGCAATGAGCGGTTC-3′; reverse sequence: 5′-AGGTCTTTGCGGATGTCCACGT-3′) was used as the internal control. Cq values were recorded, and relative gene expression was calculated using the 2^−ΔΔCq^ method, normalized to β-actin, and compared to the expression levels in control cells.

### 4.13. Statistical Analysis

All experiments were conducted in triplicate, while Western blotting, apoptosis assay, and qRT-PCR were in conducted in duplicate, and results are presented as mean ± standard deviation (SD). Data were analyzed using one-way ANOVA, followed by Tukey’s post hoc test for multiple comparisons. Statistical significance was set at *p* < 0.05. All statistical analyses were performed using SigmaPlot 14.0 and Microsoft Excel-2019.

## 5. Conclusions

This study highlights the therapeutic potential of combining doxo with the selective HDAC inhibitor Tas and PCI for osteosarcoma treatment. Our findings demonstrate that this combination therapy exerts synergistic anti-tumor effects by inhibiting cell proliferation, inducing apoptosis through caspase activation, and suppressing metastasis via MMP downregulation. Moreover, combination therapy effectively downregulated the RUNX2/pAKT signaling pathway, which is implicated in osteosarcoma progression and drug resistance, thereby enhancing the pro-apoptotic effects of doxo while mitigating its limitations. Importantly, Tas and PCI exhibited minimal cytotoxicity in normal human cells, further supporting their potential use as targeted therapeutic agents. The efficacy of this combination was validated across both 2D and 3D tumor models, underscoring its potential translational relevance. A key limitation of the current study is the primary focus on 48 h endpoints without a dedicated spheroid regrowth/recovery experiment to fully assess long-term effects. However, given the limitations of in vitro studies, further in vivo investigations are necessary to confirm these findings, optimize dosing regimens, and evaluate potential resistance mechanisms. Future preclinical and clinical studies should focus on elucidating long-term safety, pharmacodynamics, and patient-specific biomarkers that could guide personalized therapy. Overall, this study provides a strong rationale for further exploration of HDAC-targeted combination strategies in osteosarcoma, offering new avenues for improving therapeutic outcomes in patients with this aggressive malignancy.

## Figures and Tables

**Table 1:** An overview of different combination therapies using doxorubicin and HDAC inhibitors.

Combination Therapy	Cancer Type	Mechanism of Action	Key Findings	Reference
Doxo + quercetin (Encapsulated in folic-acid-modified liposomes)	Osteosarcoma	Inhibits JAK2-STAT3-PD-L1 pathway	Enhances doxo efficacy via immune modulation	[4]
Doxo + PCI-24781 (HDAC inhibitor)	Osteosarcoma	Inhibits histone deacetylases (HDACs), induces apoptosis	Enhances doxo cytotoxicity and reduces tumor cell survival	[5]
Doxo + SAHA (HDAC1 inhibitor)	Triple-negative breast cancer	Epigenetic modulation, apoptosis induction	Synergistic effect with improved in vivo efficacy	[6]
Doxo + antioxidants (targeting oxidative stress injury)	Cardiotoxicity model	Reduces doxo-induced oxidative damage	Potential cardioprotective effects	[17]
Doxo + HDAC8 inhibitor (PCI-34051) + ACY-241	Ovarian cancer	HDAC8-specific inhibition, apoptosis enhancement	Improves anti-cancer effects of ACY-241	[18]
Doxo + RUNX2 targeting therapy	Gastric cancer	Reverses p53-induced chemotherapy resistance	Overcomes doxo resistance mechanisms	[19]
Doxo + tasquinimod (HDAC4 inhibitor)	Ovarian cancer	Regulates Nur77-Bcl-2 apoptotic pathway	Increases cisplatin chemosensitivity	[20]
Doxo + vorinostat (HDAC inhibitor) + cisplatin	Osteosarcoma	HDAC inhibition, DNA damage, apoptosis enhancement	Synergistic cytotoxicity in osteosarcoma cell lines	[21]

**Table 2:** Drug treatment groups.

Treatment Groups	Dose
Control	No treatment
Doxorubicin (doxo)	3.0 µM
Tasquinimod (Tas)	10.0 µM
PCI-34051 (PCI)	10.0 µM
T1	0.1 µM Doxo + 1.0 µM Tas + 1.0 µM PCI
T2	0.1 µM Doxo + 2.0 µM Tas + 2.0 µM PCI
T3	0.5 µM Doxo + 1.0 µM Tas + 1.0 µM PCI
T4	0.5 µM Doxo + 2.0 µM Tas + 2.0 µM PCI

## Data Availability

No new data were created or analyzed in this study. Data sharing is not applicable to this article.

## Supplementary Materials

The following supporting information can be downloaded at: https://www.mdpi.com/article/10.3390/ijms26083574/s1.

## Abbreviations

The following abbreviations are used in this manuscript:

Doxo	Doxorubicin
ECM	Extracellular matrix
EMT	Epithelial–mesenchymal transition
HDAC	Histone deacetylases
MMP	Matrix metalloproteinase
PCI	PCI-34051
PBS	Phosphate-buffered saline
pAKT	Protein kinase B
PI3K/Akt	Phosphatidylinositol 3-kinase/protein kinase B
RUNX2	Runt-related transcription factor 2
Tas	Tasquinimod
