# Supplementary material for: Synergistic HDAC4/8 Inhibition Sensitizes Osteosarcoma to Doxorubicin via pAKT/RUNX2 Pathway Modulation
Source: Int J Mol Sci. 2025 Apr 10;26(8):3574. doi: 10.3390/ijms26083574 (PMC12026469; doi:10.3390/ijms26083574)
Supplement: Supplementary file 1 [file ijms-26-03574-s001.zip › ijms-3512006-supplementary.pdf]

Fig. S1A

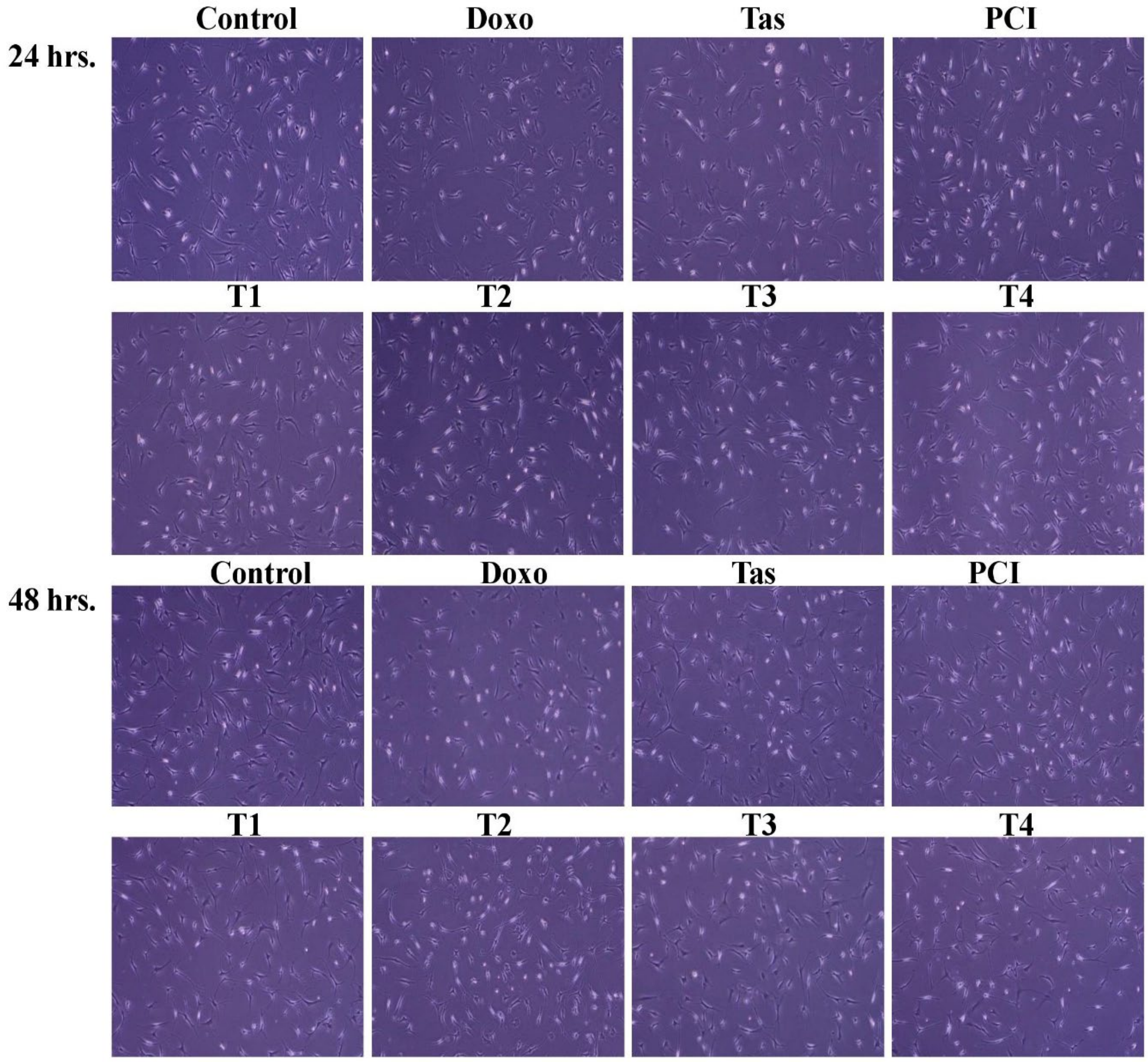

24 hrs.
Control
Doxo
Tas
PCI
T1
T2
T3
T4
48 hrs.
Control
Doxo
Tas
PCI
T1
T2
T3
T4

Fig. S1B

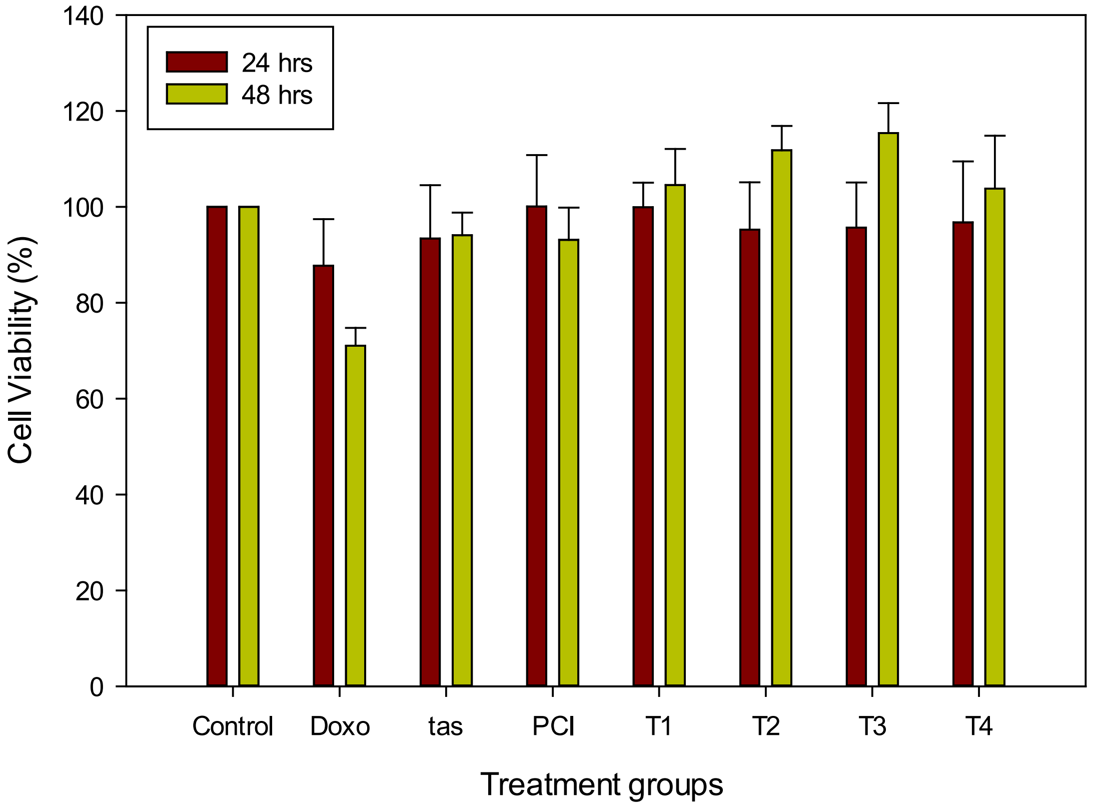

Figure S1. Tas and PCI exhibit no inhibitory effects on normal tenocyte cells. A. Morphological changes in human tenocyte cells following treatment with Tas, PCI, and combination therapy for 24 and 48 hours. **B.** Effects of Tas and PCI on proliferation of human tenocyte cells, as assessed by CCK8 assay.

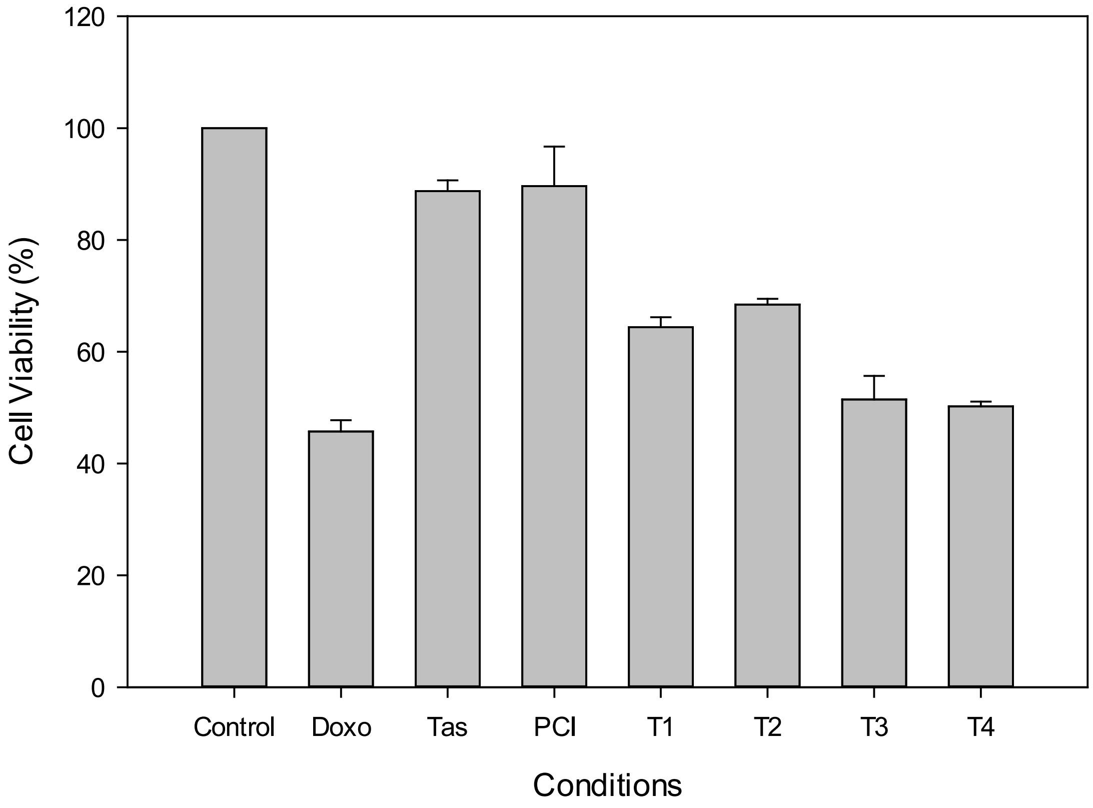

Figure S2. Inhibitory effects of doxorubicin (doxo), tasquinimod (Tas), and PCI-34051 (PCI), individually and in combination, on MG-63 osteosarcoma cell proliferation. Cell viability (CCK-8 assay): CCK-8 assay was performed after 48 hours of treatment to evaluate cell viability. This assay measures metabolic activity as indicator of cell viability and cytotoxicity. Mean values and % error are presented to compare cytotoxic effects of each treatment and combination therapy, showing their effectiveness at reducing cell viability.
